# Prostate MRI and artificial intelligence during active surveillance: should we jump on the bandwagon?

**DOI:** 10.1007/s00330-024-10869-3

**Published:** 2024-06-27

**Authors:** Vilma Bozgo, Christian Roest, Inge van Oort, Derya Yakar, Henkjan Huisman, Maarten de Rooij

**Affiliations:** 1 Diagnostic Image Analysis Group, Radboud University Medical Center, Nijmegen, The Netherlands; 2 Department of Medical Imaging, Radboud University Medical Center, Nijmegen, The Netherlands; 3 Departments of Radiology, Nuclear Medicine and Molecular Imaging, University Medical Center Groningen, University of Groningen, Groningen, The Netherlands; 4 Department of Urology, Radboud University Medical Center, Nijmegen, The Netherlands; 5 Department of Radiology, Netherlands Cancer Institute, Amsterdam, The Netherlands; 6 Department of Circulation and Medical Imaging, Norwegian University of Science and Technology, Trondheim, Norway

**Keywords:** Prostatic neoplasms, Magnetic resonance imaging, Artificial intelligence

## Abstract

**Objective:**

To review the components of past and present active surveillance (AS) protocols, provide an overview of the current studies employing artificial intelligence (AI) in AS of prostate cancer, discuss the current challenges of AI in AS, and offer recommendations for future research.

**Methods:**

Research studies on the topic of MRI-based AI were reviewed to summarize current possibilities and diagnostic accuracies for AI methods in the context of AS. Established guidelines were used to identify possibilities for future refinement using AI.

**Results:**

Preliminary results show the role of AI in a range of diagnostic tasks in AS populations, including the localization, follow-up, and prognostication of prostate cancer. Current evidence is insufficient to support a shift to AI-based AS, with studies being limited by small dataset sizes, heterogeneous inclusion and outcome definitions, or lacking appropriate benchmarks.

**Conclusion:**

The AI-based integration of prostate MRI is a direction that promises substantial benefits for AS in the future, but evidence is currently insufficient to support implementation. Studies with standardized inclusion criteria and standardized progression definitions are needed to support this. The increasing inclusion of patients in AS protocols and the incorporation of MRI as a scheduled examination in AS protocols may help to alleviate these challenges in future studies.

**Clinical relevance statement:**

This manuscript provides an overview of available evidence for the integration of prostate MRI and AI in active surveillance, addressing its potential for clinical optimizations in the context of established guidelines, while highlighting the main challenges for implementation.

**Key Points:**

- *Active surveillance is currently based on diagnostic tests such as PSA, biopsy, and imaging*.
- *Prostate MRI and AI demonstrate promising diagnostic accuracy across a variety of tasks, including the localization, follow-up and risk estimation in active surveillance cohorts*.
- *A transition to AI-based active surveillance is not currently realistic; larger studies using standardized inclusion criteria and outcomes are necessary to improve and validate existing evidence*.

## Introduction

Prostate cancer (PCa) is one of the most prevalent cancers with a broad spectrum of clinical behavior [1] with increasing trends noticed in all European countries, highlighting the need to optimize patient management. Different active treatment approaches (e.g., prostatectomy or radiotherapy) and other management options, such as active surveillance (AS), are offered depending on patient risk, categorizing them into low-risk, intermediate-risk, and high-risk prostate cancer. AS is the only management strategy for low-risk PCa patients, and one of the management options for specific intermediate-risk PCa, according to the European Association of Urology guidelines (EAU) [2]. AS focuses on providing timely management and preventing overtreatment in patients with indolent disease. Men on AS are currently closely monitored with prostate-specific antigen (PSA) measurements, repeat biopsies, digital rectal examinations (DRE), and imaging modalities to detect disease progression rather than opting for active treatment [3]. However, the diagnostic performance of the current monitoring tools [4–6] remains low.

The potential gain from further protocol optimizations based on clinical parameters and biopsy results is likely limited, prompting a look at more recent developments for further optimizations. Prostate magnetic resonance imaging (MRI) and artificial intelligence (AI) may enhance several aspects of AS protocols. However, the contributions and challenges of MRI and AI need to be clearly understood before their integration into clinical practice. This review aims to analyze AS protocols from their introduction until today and to provide an overview of diagnostic AI algorithms for AS. In addition, we offer practical recommendations on addressing some of the challenges prior to the implementation of AI AS in the clinical setting.

## Active surveillance with MRI

Eligibility for AS is determined based on risk categories as defined in guidelines. This risk stratification is based on PSA levels, Gleason scores, and the clinical stage of DRE. EAU guidelines provide two strong recommendations with regard to AS [2]. First, to include a strict follow-up protocol based on DRE (at least once yearly), PSA (at least once every 6 months) with repeat biopsies every 2 to 3 years [2]. Second, it recommends performing an MRI and repeat biopsy if PSA is rising (PSA-doubling time < 3 years) [2]. MRI can contribute in several ways in AS protocols including patient selection, MR-targeted biopsies, and serial imaging monitoring.

The value of MRI and targeted biopsies was proven during the ASIST trial; a prospective, multicenter, randomized, open-label trial with low-risk (Gleason grade group (GG) = 1, PSA < 10 ng/mL, clinical stage ≤ T2b) prostate cancer men managed with AS [7]. The trial investigated the proportion of men progressing to GG ≥ 2 in men receiving MR-targeted biopsies and systematic biopsies at baseline [7]. Two-year follow-up revealed approximately 50% more AS failure cases or disease progression in the systematic biopsy group compared to the MRI group (35% vs 19%, *p* = 0.017) [8], highlighting the role of MRI in the inclusion of men for AS.

MRI provides a non-invasive way of monitoring patients during AS. To standardize reporting from cohort studies involving men on active surveillance for PC, the European School of Oncology convened the Prostate Cancer Radiological Estimation of Change in Sequential Evaluation (PRECISE) panel to develop recommendations [9]. The PRECISE recommendations were initially introduced in 2016 and reiterated in 2024 with the presentation of their second version [10]. This standard recommends that radiologists should report on patient characteristics, MR details, the likelihood of the clinical stage, clinically significant prostate cancer (csPCa), the imaging sequence where the lesion can be best seen, and also categorize MRI-visible lesions using either PI-RADS (Prostate Imaging-Reporting and Data System) or a Likert score [11, 12]. This ensures standardized data collection, reporting, and lays the foundation for future guideline recommendations [13].

PRECISE assigns a 1-to-5 score to the likelihood of progression on serial MRI examinations. The system incorporates changes in the conspicuity of suspicious features, the lesion volume, seminal vesicle or nodal involvement, extracapsular extension, bone metastasis in the baseline and follow-up MRI examinations. Preliminary results from current studies where radiologists use the PRECISE standard to monitor progression in AS cohorts reveal an AUC (area under the curve) performance of approximately 0.84 [5, 6].

The standard for reporting used by radiologists to assess prostate MRI is PI-RADS [14]. While the PI-RADS guidelines are not designed to assess lesion progression [14], Roest et al [4] reported the radiologists’ performance in detecting PCa progression using this standard reached an AUC of 0.69. Notably, this performance was lower than that of the AI, highlighting the need for an improved diagnostic tool for serial MRI.

## Active surveillance with MRI and AI

AI could have a potential role in addressing some of the limitations of the use of MRI during active surveillance in different ways, such as by streamlining serial MRI assessments, decreasing MRI inter-observer variability, and assisting non-expert readers [15]. Current studies explore AI techniques such as machine learning and deep learning to improve AS. Machine learning classifiers mainly employ clinical data and radiomic features of the MR scans at consecutive time points [6, 16]. Radiomics represents the extraction of quantitative texture measurements and patterns from MRI [16] that cannot be acquired by the human eye [17]. Deep learning methods can automatically learn to perform various diagnostic tasks from large, annotated medical imaging datasets. Both radiomics [18, 19] and deep learning models [1, 20] have demonstrated a high performance in the detection and assessment of PCa. The MRI AI studies in AS cohorts can be divided into two groups based on their primary task, namely, lesion detection and localization, and detection of disease progression.

### Lesion detection and localization

The first group of studies focuses on lesion detection and localization in AS cohorts. While the PI-RADS scoring system has standardized the detection and characterization of lesions on prostate MRI, it is associated with variation between readers. Inadequate characterization of prostate lesions could lead to an underestimation of true histopathology and could falsely identify patients as negative or benign. AI-driven lesion detection and classification in prostate MRI promises enhanced accuracy in reading MRI exams, with improved interobserver agreement and comparable diagnostic accuracy to radiologists for the detection of csPCa [21].

In the context of AS, enhancing the specificity of prostate MRI readings through AI would be desirable to enable a reduction in repeat biopsies. Arber et al [22] trained a radiomic region-of-interest-based computer-aided diagnosis (CAD) system that uses the wash-in rate and quantitative image parameters to define the risk that a lesion corresponds to a GG ≥ 2 cancer in the peripheral and transition zone, respectively. Both the CAD score and the PI-RADS score determined by the genitourinary radiologist reached an AUC of 0.81 for the detection of upgrading to clinically significant PCa in a cohort of 186 patients while under AS. Combining CAD findings and PSA density (PSAd) to trigger baseline biopsy would have avoided 47/184 (26%) biopsies while missing 3/51 (6%) GG = 2 cancers and no GG ≥ 3 lesions.

On the other hand, Oerther et al [21] tested the Siemens Healthcare CE-certified deep-learning software MR Prostate AI on an AS cohort of 56 patients. For the detection of upgrading to clinically significant PCa at MRI, a high sensitivity and moderate specificity of 96% and 25%, respectively, were reached.

### Monitoring disease progression

The second group of studies assessed the possibility of monitoring disease progression in patients with AS. These studies included radiomics, machine learning, and to a lesser extent, deep learning-based approaches. Table 1 summarizes the characteristics of recent studies employing AI to monitor the progression of patients in AS cohorts.

**Table 1:** Prostate cancer active surveillance AI studies monitoring disease progression

Author [Reference]	Participants	AI Method	Inclusion Criteria	AS Progression Definition		AUC (95% CI)
				Radiological Progression/Clinical Variables	Pathological Progression	
Sushentsev et al [5]	76	TSR framework predicting histopathological PCa progression from longitudinal changes in radiomic features.	GG 1&2 disease with ≤ 10% Gleason pattern 4.	PRECISE ≥ 4	Biopsy-proven GG upgrade	0.86 (0.78–0.94)
Roest et al [4]	73	DL model & SVM using clinical variables and likelihood scores to predict the likelihood of csPCa at current examination.	Patients with PIRADS ≥ 2 lesions without prior diagnosis of csPCa.		GG > 1	0.86 (0.77–0.93)
Sushentsev et al [24]	332	Machine learning for longitudinal predictive modelling of PCa progression using PSA.	Men aged 50–80 with GG 1–2, < 50% core involvement, ≤ 2-core Gleason pattern 4, clinical stages T1–T2, PSA ≤ 20 ng/mL, and fit for radical treatment options.	PRECISE = 5	Biopsy-proven GG upgrade	0.74
Midya et al [16]	50	Radiomic feature classifier using baseline and delta radiomics features and clinical variables.	Patients with histopathologically documented PCa enrolled on AS, 3-T MRI and systematic biopsy at baseline, PSA measurements and biopsies at least every 12 months for ≥ 3 years, including at least one additional 3-T MRI between 18 and 36 months.	PSA, tumor volume, age	Increase in GG or the number of positive cores	0.84
Sushentsev et al [23]	70	Binary classifiers using different machine learning techniques.	Men (50–80 years) suitable for radical therapy with histologically proven prostate adenocarcinoma, clinical stages T1–T2, PSA ≤ 20 ng/mL, GG ≤ 2, < 50% overall core involvement.	PRECISE = 5	Confirmed histopathological progression	0.75 (0.64–0.86)
Sushentsev et al [6]	64	ML methods using radiomics features and clinicopathologic variables.	Patients with biopsy-proven PCa enrolled in the local AS program with a minimum follow-up of 2 years, with their first and last 3-T MRI scans performed on the same magnet and at least one repeat targeted biopsy performed within a year of the last MRI.		Histopathological progression on repeat targeted biopsy	0.82
Roest et al [25]	875	Three end-to-end DL models comparing two sequential MRI scan to detect progression to csPCa.	Patients with at least two consecutive biparametric MRI scans of the prostate between 2014 and 2021.		GG ≥ 2	0.72 (0.72–0.81)

*PI-RADS* prostate imaging-reporting and data system, *PRECISE* prostate cancer radiological estimation of change in sequential evaluation, *GG* gleason grade group, *TSR* time series radiomic, *DL* deep learning, *SVM* support vector machine, *PC a*prostate cancer, *PSA* prostate-specific antigen, *ML* machine learning, *csPCa* clinically significant prostate cancer, *AS* active surveillance, *MRI* magnetic resonance imaging

Midya et al [16] explored radiomic patterns and clinical variables such as PSA and tumor volume at diagnosis in a machine-learning random forest classifier to assess progression. The highest AUC of 0.84 ± 0.20 was reached when incorporating the difference in radiomic features and clinical variables.

Sushentsev et al [23] proposed several machine learning models using baseline radiomic features and clinical variables comprising PSA, MRI-derived gland volume, PSA density, MRI-derived Likert score of tumor suspicion, target lesion localization, and target lesion biopsy grade group to predict baseline risk of PCa progression on AS. Their best model reached an AUC of 0.75 (95% CI 0.64–0.86) by combining clinicopathological predictors with T2WI-derived radiomic features. Similar results were reached by the same group [24] also when using PSA density in a recurrent neural network for dynamic monitoring of the risk of progression.

Using the same model, this group [5] has also explored integrating both imaging and clinical data, including T2W and apparent diffusion coefficient map radiomic features, PSA and the derived PSA density, from multiple time-points to monitor AS patients. The reached performance of 0.86 was significantly higher than when using radiomic features and PSAd only from baseline and final scans. This suggests that MRI-based AI has the potential to function as an additional risk stratification tool, and could aid in selecting appropriate candidates for rebiopsy based on changes in lesion characteristics.

In another study by Sushentsev et al [6], the performance of several machine learning classifiers using radiomic features and clinicopathological characteristics, namely age, PSA, gland volume, PSA density, follow-up period, biopsy grade groups, and location of the target lesion in percentage, were compared to PRECISE. The most optimal machine learning classifier was a parenclitic network with an AUC of 0.82. However, the differences in performance between the ML algorithms and PRECISE were non-significant. PSA and PSAd were significantly higher in progressors compared to non-progressors [6].

Roest et al [4] reached a similar performance of 0.86 using a hybrid approach incorporating a deep learning model for lesion detection, followed by a support vector machine classifier to detect PCa progression.

In another study by Roest et al [25], an end-to-end deep learning model based on sequential MRI was used to detect progression to csPCa at follow-up in patients without csPCa at baseline. The addition of prior imaging to the model and clinical parameters resulted in an improved performance of 0.76 AUC compared to 0.73 AUC for the single timepoint baseline.

Overall, we observed that AI algorithms’ performance in each of these studies improved when information about the patients’ clinicopathological characteristics and their changes between different time points was added. This highlights the potential of AI in this task when additional data are provided to the models. However, because of the low number of participants in these studies, we should be critical when analyzing and referring to these results.

## Active surveillance AI challenges before the implementation in the clinical practice

We noted that AI achieves a promising performance in current studies. This can further improve AS protocols in the future. Despite this, we have identified several challenges that need to be addressed before AI can be considered as an addition to AS guidelines.

### Progression definition

The first observation is that there is an ambiguity regarding the primary criterion for pathologic progression. The focus of pathologic progression is on the increase of the GG score. More specifically, the detection of GG > 1 lesions in patients with previously detected GG = 1 lesions (Table 1). However, contemporary guidelines do not always recommend a transition to active treatment when Gleason pattern 4 is detected [9, 10]. As a result, the use of ISUP upgrades as an endpoint may result in systems that produce false positives in patients with favorable conditions, which could lead to unnecessary exclusions from AS. However, given that most AI studies rely on retrospective data, historical datasets may lack the necessary information to ascertain if a progression criterion was met. Additionally, adherence to earlier versions of guidelines may have led to patients discontinuing AS when current guidelines would advise otherwise, resulting in incomplete datasets. Nevertheless, we encourage AI researchers to develop their models on endpoints, aligning as closely as possible with discontinuation criteria defined in AS guidelines.

The second observation is that several studies evaluated their models to the PRECISE standard which defines a score ≥ 4 as constituting radiological progression [9, 10]. However, there is an ongoing discussion on the optimal method for measuring lesion size, that allows for discrimination between true PCa progression and benign changes (natural fluctuations in tumor volume and measurement errors) [10, 15], and a specific threshold for changes in size and conspicuity. Thus, additional data and collaboration between radiologists, urologists, and pathologists are necessary to define adequate and unified methodologies for measuring lesion size and set robust thresholds.

Overall, there is a heterogeneity in the specific criteria employed to define progression, which can be attributed to the use of different guidelines available. Moreover, guidelines evolve over time, further complicating the standardization of efforts. The diversity in inclusion criteria presents challenges in the comparison of reported diagnostic accuracies and calls into question their applicability across institutions with differing standards. These issues are likely to persist, pending the standardization of AS guidelines. However, to facilitate diagnostic accuracy comparisons, authors may include evaluations for alternative progression criteria as secondary outcomes, where possible.

### Heterogeneity in inclusion criteria

AI studies varied widely in their inclusion criteria, which raises concerns about the applicability of trained models across different clinical settings and protocols. A model developed for one population may not necessarily perform well in a different clinical context with different inclusion criteria. Therefore, it is essential to assess a model’s predictive value in a specific population before deploying it. Despite these differences, populations across protocols generally share similarities, and AI models have the potential to generalize if trained on large and diverse datasets. Ultimately, it is crucial for cohort inclusions to closely resemble real-world clinical practice to ensure realistic and applicable results.

### Lack of available datasets

The first constraint observed in existing AI algorithms for AS in PCa patients is the limited sample size and the lack of validation in multi-center data. Table 1 illustrates that the majority of the studies have sample sizes under 100 participants. This limitation arises from the stringent inclusion criteria demanding the presence of MR-visible lesions which represents a criterion that only makes up around 50% of patients in AS cohorts [26]. Other contributing factors include MRI acquisition protocols, and the ground truth assessment [5]. In addition to this, there are currently no freely available open-source AS MRI datasets that can be used for algorithm development. Datasets used in the available studies are covered by data transfer agreements, confidentiality agreements with the patient, or should be requested from the authors following the respective institutional guidelines. Due to this lack of sufficiently sized datasets, the available studies are often limited to simplistic radiomics models, while, in high-resource settings, such as the initial detection of PCa, deep learning techniques have demonstrated superior performance over radiomics. With more data, we may expect an increased use of deep learning, with increasing diagnostic performances on a larger scale. Therefore, complying with open science practices and FAIR principles (publicly available datasets, open-source models) is essential for the continual validation of algorithms and the development of more robust models.

### MR image quality

MR image quality plays an important role during the AS programs, influencing the entire radiological pathway, from the initial diagnosis to the continuous monitoring of the disease. Optimal image quality provides clear and detailed information about the size, image features, and location of prostate lesions that is needed to make informed decisions on accurate patient risk stratification and monitoring progression. Adhering to PI-RADS minimal technical recommendations does not guarantee optimal image quality. The quality of the images is influenced not only by hardware factors but is also influenced by patient-related factors (such as movement) and patient preparation. The recently updated Prostate Imaging Quality (PI-QUALv2) [27] is the proposed scoring system for a standardized assessment of MR image quality relying on a set of subjective criteria [28].

Additionally, image quality influences the training and validation of AI algorithms. Images of suboptimal diagnostic quality containing low spatial resolution, reduced signal-to-noise ratio, motion, or prosthesis artifacts can hinder training, validation, and the performance of the AI in testing cohorts. These algorithms usually rely on high-quality data for tasks such as segmentation, registration, prognosis, etc [29]. Therefore, standardized MRI acquisition protocols across centers and rigorous quality control standards such as PI-QUALv2 [27] are crucial to obtaining reliable datasets for robust AI development and generalizability of the AS models [29].

### Radiologist performance

There is currently a knowledge gap concerning the performance of the radiologist while monitoring patients on AS. While PI-RADS is the standard that is used for the detection of csPCa lesions, its main task is not surveillance but rather the initial assessment of an MRI. This standardized assessment lacks criteria for reporting on sequential MRIs [14]. For this purpose, the PRECISE system offers radiologists a standardized approach. Using PRECISE consistently in sequential MRIs could offer a more stable benchmark performance for AI. Future studies about AI in AS should include a comparison of the algorithm performance with that of radiologists using PI-RADS and PRECISE.

## Conclusion

It seems currently infeasible to jump on the prostate MRI AI for AS bandwagon. AI for AS based on MRI may improve several aspects in the future, but its application still remains underexplored. More efforts will be required to establish standardized inclusion criteria and a progression definition of AS. In addition, increasing the inclusion of scheduled MRI examinations in AS protocols is expected to increase the availability of larger datasets to facilitate future research. This will allow for training the AI for AS in much larger datasets and validating the results.
